# An Assessment of the Cyto-Genotoxicity Effects of Green-Synthesized Silver Nanoparticles and ATCBRA Insecticide on the Root System of *Vicia faba*

**DOI:** 10.3390/nano15010077

**Published:** 2025-01-06

**Authors:** May A. Al-Saleh, Hanan F. Al-Harbi, L. A. Al-Humaid, Manal A. Awad

**Affiliations:** 1 Botany and Microbiology Department, College of Science, King Saud University, Riyadh 11451, Saudi Arabia or mayalsaleh0@gmail.com (M.A.A.-S.); lalhumaid@ksu.edu.sa (L.A.A.-H.); 2 King Abdullah Institute for Nanotechnology, King Saud University, P.O. Box 2455, Riyadh 11451, Saudi Arabia

**Keywords:** cardamom, cytotoxicity, insecticide, genotoxicity, silver nanoparticle, *Vicia faba*

## Abstract

We aimed to synthesize silver nanoparticles (AgNPs) using *Elettaria cardamomum* (cardamom) extracts and assess the cytotoxicity and genotoxicity of the cardamom extract, *cardamom*–AgNPs, and the insecticide ATCBRA—commonly used for pest control—on the root system of *Vicia faba* (broad bean). The chemical composition of the aqueous cardamom extract was identified and quantified using GC-MS, revealing a variety of bioactive compounds also present in cardamom essential oil. These included α-terpinyl acetate (21.3–44.3%), 1,8-cineole (10.7–28.4%), and linalool (6.4–8.6%). The successful green synthesis of AgNPs was confirmed through various micro-spectroscopic techniques, including UV-Vis spectroscopy, transmission electron microscopy (TEM), and energy-dispersive spectroscopy (EDS). UV-Vis analysis showed a strong peak between 420 and 430 nm, indicating the presence of AgNPs. TEM imaging revealed that the synthesized *cardamom*–AgNPs were monodispersed, primarily spherical, and semi-uniform in shape, with minimal aggregation. EDS analysis further confirmed the composition of the nanoparticles, with *cardamom*–AgNPs comprising around 60.5% by weight. Cytotoxicity was evaluated by measuring the mitotic index (MI), and genotoxicity was assessed by observing chromosomal aberrations (CAs). The roots of *Vicia faba* were treated for 24 and 48 h with varying concentrations of ATCBRA pesticide (0.1%, 0.3%, 0.5%, and 0.7%), aqueous cardamom extract (3%, 4%, 5%, and 6%), and green-synthesized *cardamom*–AgNPs (12, 25, and 60 mg). The cytogenetic analysis of MI and CA in the meristematic root tips indicated an improvement in the evaluated parameters with the cardamom extract. However, a marked reduction in mitotic activity was observed with both ATCBRA and *cardamom*–AgNP treatments across both time points, highlighting potential cytotoxic and genotoxic effects.

## 1. Introduction

In agriculture, developing safe and efficient biocontrol agents with alternative modes of action is crucial, as the excessive use of chemical pesticides often has harmful effects on various organisms, including humans [1]. Alternatives such as insect growth regulators (IGRs), botanical compounds, and microbiological products have shown promise as replacements for conventional pesticides. Biopesticides in particular represent a modern approach to pest control. Plants naturally produce secondary compounds—such as alkaloids, terpenoids, phenolics, and flavonoids—that exhibit insecticidal properties. These compounds can disrupt key metabolic pathways in insects, leading to rapid mortality [2,3]. Despite increasing interest, botanical insecticides still represent only a small percentage of global crop protectants (Houghton, 1996) [4,5,6,7].

Several studies have demonstrated the potential of plant extracts in pest management. For example, four botanical oils—of *Melissa officinalis*, *Borago officinalis*, *Laurus nobilis*, and *Carapichea ipecacuanha*—were tested on the red palm weevil (*Rhynchophorus ferrugineus*), all of which significantly impacted the insect’s biology [8]. Similarly, El Namaky et al. [9] showed that *Punica granatum* peel extract could serve as an effective biopesticide against both the adult and larval stages of red palm weevils.

Cardamom (*Elettaria cardamomum*), a highly prized spice from the Zingiberaceae family, is noted for its rich chemical composition, which includes numerous beneficial functional groups such as hydroxyl, carbonyl, and amino acids. These compounds boost natural antioxidant levels [10]. The essential oil extracted from cardamom seeds has been found to be toxic to tomato leaf miners and is characterized by its strong, distinctive scent [11,12]. In a toxicity study, powdered cardamom seeds were shown to cause a 93% mortality rate in red palm weevils after just one day, and one of 100% after two days [13].

Nanotechnology offers significant potential for the development of new, environmentally friendly insecticides. According to Chinnamuthu and Boopathi [14], the use of nanotechnology in biocontrol could provide cost-effective solutions to many environmental challenges. The plant-mediated biosynthesis of nanoparticles, in particular, is advantageous because it is both eco-friendly and cost-effective. AgNPs synthesized through plant extracts have gained considerable attention due to their unique properties and wide range of applications, including their antioxidant, antibacterial, antifungal, antiviral, and anti-inflammatory effects and growth-promoting properties, which are highly advantageous for seed germination and early plant development. Furthermore, silver nanoparticles are a popular choice in green synthesis because of their cost-effectiveness and ease of production [15]. Research by Prażak et al. highlights the dual effects of silver nanoparticles on plants and organisms, influenced by factors like genotype, application method, concentration, size, and coating material. AgNPs are valued for their antimicrobial properties, effectively protecting crops from fungal diseases. They also promote seed germination and growth in crops like maize, watermelon, and zucchini, though some negative effects, such as inhibited maize root elongation, have been noted. Additionally, AgNPs enhance tolerance to abiotic stresses like salinity, chilling, and heat, improving resilience in plants [15].

Cytotoxicity and genotoxicity are commonly assessed using the mitotic index, which measures the proportion of cells undergoing division [16,17]. Chromosomal aberrations (CAs), on the other hand, serve as biomarkers for genotoxicity caused by various substances [18,19]. Since both human and plant root cells share similar chromosomal regulation mechanisms, plant models are widely used to study environmental mutagens and contaminants. Species such as *Allium cepa* and *Vicia faba* have long been used in clastogenicity tests due to their suitability for detecting chromosomal abnormalities [20,21]. These plant-based bioassays are highly regarded for evaluating environmental quality and DNA damage caused by chemical agents.

In the present study, we explore the synthesis of silver nanoparticles using *E. cardamomum* aqueous extracts and assess the cytotoxicity and genotoxicity of cardamom extracts, *cardamom*–silver nanoparticles, and the insecticide ATCBRA against the root system of *Vicia faba*. To the best of our knowledge, this study is the first to evaluate the cytotoxic and genotoxic effects of cardamom extract and synthesized cardamom–AgNPs using a plant model.

## 2. Materials and Methods

### 2.1. Vicia faba Seed Germination

The College of Food and Agricultural Sciences at King Saud University provided healthy *V. faba* seeds for the study. The germination process started with a brief 3 min sanitization using 5% chlorine, followed by a 12 h soak in water. After soaking, the seeds were wrapped in moist absorbent cotton and rolled until the radicles reached 1 to 2.5 cm in length. Once germinated, the seeds were transferred to Petri dishes containing different concentrations of cardamom nanoparticles, cardamom extract, and conventional insecticide. They were left at room temperature for 24 and 48 h.

### 2.2. Elettaria cardamomum Aqueous Extract Preparation

Cardamom (*Elettaria cardamomum*) seeds were acquired from a local market. The extraction protocol of Kaushik et al. [22] was used with modifications. Ten grams of freshly ground cardamom seeds was soaked in 100 mL of double-distilled water. After that, the mixture was put on a Velp F20500050 Digital Ceramic Stirring Hot Plate (VELP Scientifica Srl—Usmate Velate—Italy), which had a 250 V timer, and was stirred for 30 min. The suspension was then moved to an MPR-721-PE pharmaceutical refrigerator (Panasonic Healthcare Co., Ltd.—Gunma—Japan) and kept there overnight. The extract solution was first filtered using two to three layers of gauze. The suspension was then centrifuged using a HERMLE Z 206 A centrifuge (HERMLE Labortechnik—Gosheim—Germany) for 2 min at a speed of 1000 rpm. The supernatant was collected, and the resulting residue was disposed of. The cardamon extract was now ready for additional analyses. Gas chromatography–mass spectrometry (GC-MS, gas chromatograph–mass spectrometer 7890B GC system—Agilent—Santa Clara, CA, USA) was used to analyze the sample.

### 2.3. Synthesis of AgNPs Utilizing Aqueous Extract of Elettaria cardamomum

To synthesize *cardamom*–AgNPs containing cardamom extract using a modified protocol [23], three distinct concentrations of silver nitrate (AgNO_3_) (1.77, 3.68, and 8.83 mM) were carefully weighed in relation to the extract. Subsequently, double-distilled water was added to each concentration, and the mixtures were covered with aluminum foil and placed on a stirring hot plate. The presence of bubbles during the heating process indicated that the solutions had reached their boiling points. Next, 5 mL of *E. cardamomum* aqueous extract was progressively added to the wall of the beaker. Depending on the concentration, the solution changed to a pale yellow color that gradually darkened. The mixture was left to rest for 30 min. It is crucial to note that the efficacy of the extract lasts for only one to two days.

A wide range of nanoparticle characterization techniques were employed, including spectroscopic, separation, and microscopic methods. The most commonly employed, accessible, and efficient technique for the preliminary confirmation of nanoparticle formation and determining their characteristics is ultraviolet–visible (UV-Vis) spectroscopy. Using the Shimadzu UV-1800 UV-Vis spectrometer—Kyoto—Japan, we were able to obtain the absorbance spectra of the colloidal sample between 200 and 950 nm. Distilled water served as the control. AgNPs were examined using a JEOL JSM-7610F—Tokyo—Japan microscope equipped with an energy-dispersive X-ray spectroscopy (EDX) attachment. In addition, the AgNPs’ size, shape, and morphology were all assessed by transmission electron microscopy (TEM), which was conducted on a JEOL JEM-1400Plus—Tokyo—Japan electron microscope working at 100 kV. The TEM grid was created by applying a droplet of the bio-reduced diluted solution to a copper grid coated in carbon and then using light to dry it. The narrow size distribution and monodispersity of the *cardamomum*–AgNPs were further confirmed using the polydispersity index (PDI) and were measured via dynamic light scattering (DLS) with the HT Laser ZEN3600 (Malvern Nano Series, Malvern, UK). Prior to particle size analysis, the sample suspensions were sonicated to ensure uniform dispersion and prevent aggregation.

### 2.4. ATCBRA EC Pesticide Concentration Preparation

The active ingredients of ATCBRA EC insecticide are quinalphos (20%) and cypermethrin (3%). Quinalphos is one of the most commonly used organophosphate insecticides, and cypermethrin is a pyrethroid compound that is widely used due to its high insecticidal potential and slow onset of resistance in pests. It is used to treat the insect pests of several plants, including legumes.

Different concentrations of ATCBRA EC pesticide were prepared by adding 0.1, 0.3, 0.5, and 0.7 mL of ATCBRA EC pesticide with a micropipette, and then adjusting the volume to 100 mL with distilled water. The treatments were then ready for experimental use under two separate durations (24 h and 48 h).

### 2.5. Cytological Examination

The treated roots were placed in beakers, to which 1 N HCl and a laboratory thermometer were added, and then transferred into a GFL 1002 water bath (Gesellschaft für Labortechnik mbH (GFL)—Burgwedel—Germany) for 10 min or until the roots become soft and able to be dyed. They were then washed with distilled water, dyed with a Fuchsine dye, and examined under a digital Leica DM750 microscope (Leica Microsystems GmbH—Wetzlar—Germany). To capture the field of view for each treatment, the microscope was equipped with a camera. Two programs, Picasa 3 and LAS EZ, were used for the visual analysis. Three replicates were used for each treatment and scoring were conducted. A minimum of 1000 mitotic cells were selected randomly and counted from each slide.

#### 2.5.1. Mitotic Index (MI)

MI was calculated as the average number of 1000 dividing cells from different root tips for each treatment using the following equation:

% of mitotic index (MI) = (No. of dividing cells)/(Total No. of cells examined) × 100

where:

Total Number (No.) of cells = number of dividing cells + number of non-dividing cells.

#### 2.5.2. Chromosomal Aberrations (CA)

The percentage of total abnormalities was calculated as follows:

% of total abnormalities = (No. of abnormal cells × 100)/(Total No. of dividing examined cells).

### 2.6. Statistical Analyses

The experiment was conducted in triplicate. Data on the ratios of chromosomal aberrations and the mitotic index were collected. The data were presented as the mean value plus or minus the standard error (SE). The Whitney U-test was used to analyze the data, and a significance level of *p* < 0.05 was used to determine statistical significance.

## 3. Results and Discussion

### 3.1. Characterization of Elettaria cardamomum Aqueous Extract

**Figure 1:**
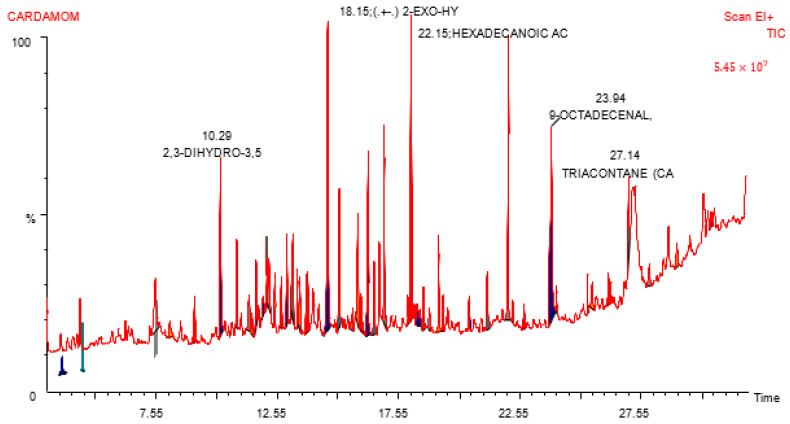
Chromatograms obtained from GC-MS screening of aqueous extracts of *Elettaria cardamom* identified by GC-MS/MS.

The identification and quantification of the chemical components of cardamom aqueous extract were conducted using GC-MS, which confirmed the presence of numerous compounds (Figure 1). Ansari et al. [24] and Alam et al. [25] previously reported that the predominant compound identified in this extract was trisdibutylphenyl phosphate, constituting 48.19% of the extract at a retention time of 31.278. In the NIST compound library, it exhibited a 93% similarity index with the chemical formula C_42_H_63_O_3_P. The second most abundant compound was nonadecane, accounting for 6.04% at a retention time of 13.999, and showing a 96% similarity index with the chemical formula C_19_H_40_. Cetane ranked third in abundance, with 4.55% at a retention time of 11.784 and a similarity index of 97%. Other compounds included clionasterol (2.84%), heneicosane (2.58%), glycidyl oleate (2.51%), and anozol (2.27%). Many of these major phytochemical compounds possess pharmacological activity or have valuable applications in various industries.

Numerous studies have highlighted the bioactive components present in cardamom essential oil, such as α-terpinyl acetate (21.3–44.3%), 1,8-cineole (10.7–28.4%), and linalool (6.4–8.6%). Green cardamom essential oil analysis using GC-MS identified twenty-six compounds, with α-terpinyl acetate (38.4%), 1,8-cineole (28.71%), linalool acetate (8.42%), sabinene (5.21%), and linalool (3.97%) acting as major bioactive components. Essential oils are complex mixtures comprising over 100 volatile lipophilic molecules, responsible for the distinctive flavor, scent, and aroma of plants. In cardamom capsule and seed essential oils, 1,8-cineole, limonene, and α-terpinyl acetate contribute significantly to the flavor profile. Additionally, cardamom oil contains essential flavoring elements like α-pinene, β-pinene, terpineol, citronellal, linalool, and allo-aromadendrene [26,27].

### 3.2. Cardamom–AgNP Characterization

**Figure 2:**
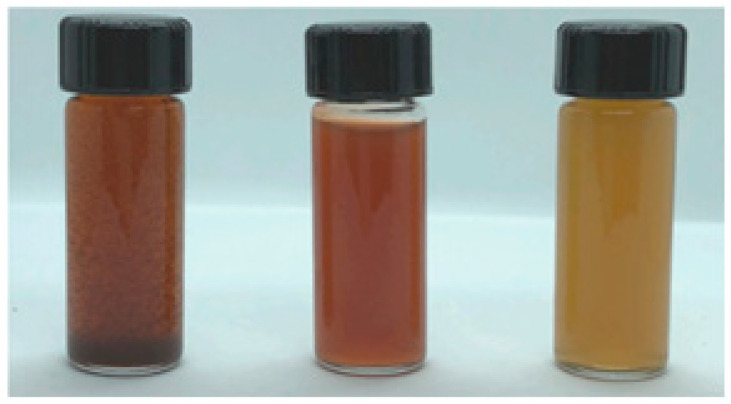
The color conversion of reaction mixtures into cardamom–AgNPs (60, 25, and 12 mg, respectively).

Only a few minutes after adding the aqueous cardamom extract, the reaction mixture changed color. As soon as cardamom–AgNPs began to form and nucleate, the reaction mixture turned brown, ranging from shades of light to dark brown (Figure 2). This hue may have been produced by surface plasmon vibration stimulation in the metal nanoparticles, and the conversion of Ag^+^ ions into Ago by the aqueous extract. Furthermore, nanoparticle size determines the wavelength at which surface plasmon resonance of AgNPs is at its maximum absorption (λ_max_), hence determining the color of the AgNPs [28].

**Figure 3:**
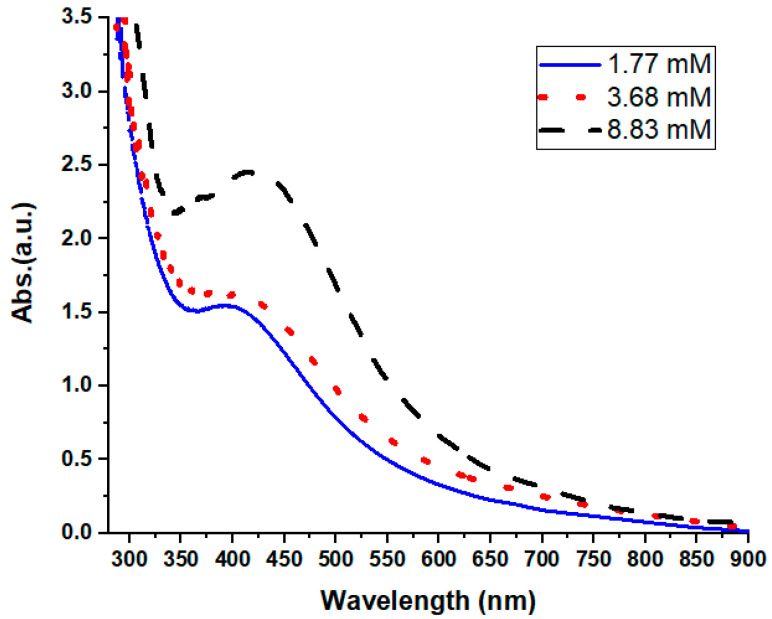
UV spectra of synthesized AgNPs using three distinct concentrations of AgNO_3_.

One useful method of elucidating how metal nanoparticles in water originate and how stable they are is UV-Vis spectroscopy. In this study, the absorbance spectra of the colloidal material were recorded within the 200–800 nm range. Figure 3 shows that data were acquired showing the presence of AgNPs, with a wide and robust peak between 420 and 430 nm (the typical absorption band for AgNPs is 420 nm). Absorbance gradually decreases as the wavelength shifts in the absorption spectra with decreasing concentrations of silver precursor [29]; congruent with this, Figure 3 also shows a shrinkage of the sharpness of the peak. Moreover, as noted by Zhang et al. [30], a reduction in bandwidth is indicated by the intensification of the plasmon bands. Our UV-Vis spectra results indicate that the extract contained components that facilitated the production of the AgNPs, and AgNO_3_ played a crucial role in this process. As the concentration of AgNO_3_ increased, so did the intensity of the absorption. Furthermore, at low concentrations, the surface plasmon peak was at 402 nm; at large concentrations, it gradually shifted to a higher wavelength of 426 nm. Depending on the size and form of the particles, this might have been a blue shift [31,32]. This band can be attributed to the absorption of colloidal AgNPs in the 400–430 nm range by surface plasmon vibration stimulation, as stated by Widatalla et al. [33]. Therefore, further analysis was conducted to make use of the high-concentration sample.

One reason for the significant interaction between light and metal surfaces is that, when excited by light at certain wavelengths, conduction electrons on such surfaces undergo a collective oscillation. SPR is an absorption band that is created when free electrons in metal nanoparticles vibrate in resonance with light waves. According to Anandalakshmi et al. [34], the occurrence of the peaks indicates the SPR capabilities of AgNPs. The optical characteristics of AgNPs, including their absorption, transmission, reflection, and emission of light, are very sensitive to changes in the material and can vary greatly from those of the bulk material. Figure 3 clearly demonstrates that the sample AgNPs had a robust absorption peak. Since AgNPs have a high absorption peak in the UV region as a result of surface plasmon excitation, the absorption spectra acquired by UV-Vis spectroscopy were accurate in establishing their existence. A comprehensive analysis of a component’s chemical, physical, and biological characteristics is necessary to develop and manufacture nanoparticles with the appropriate sizes, shapes, and functionalities [35].

**Figure 4:**
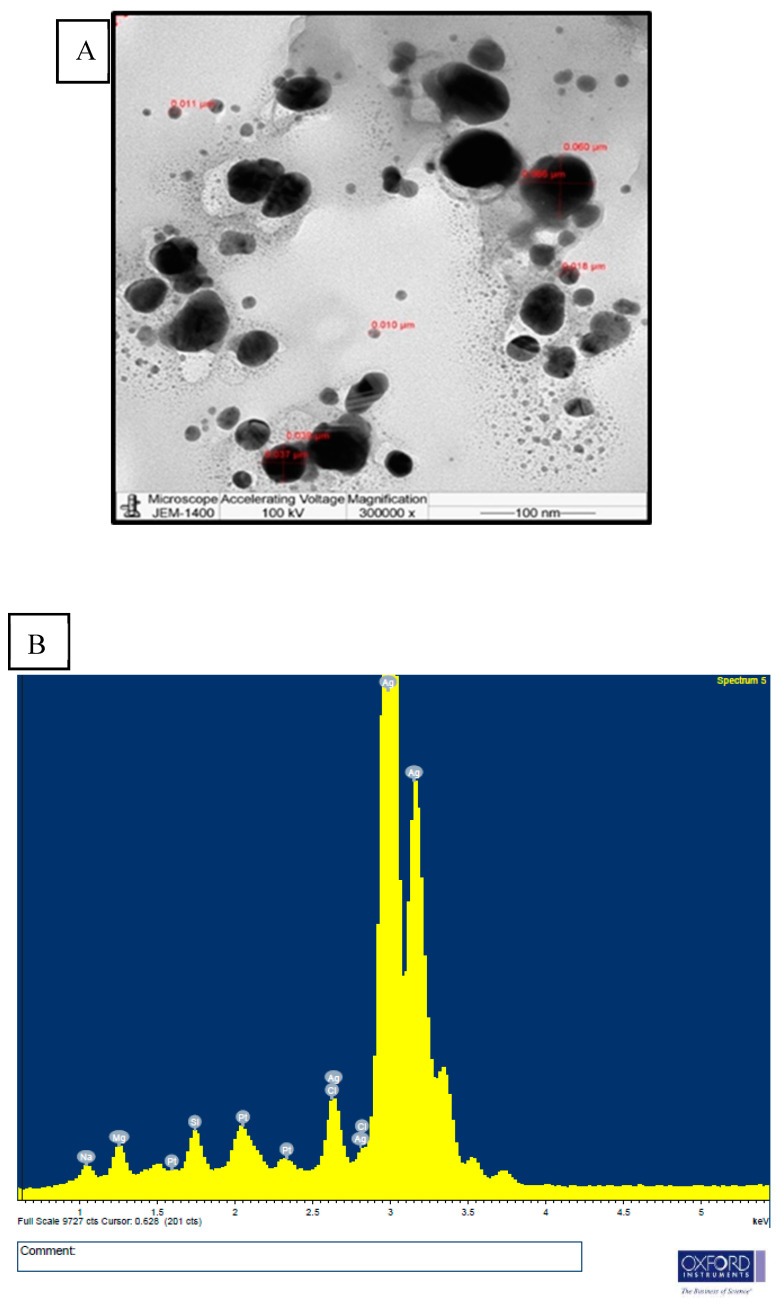
(**A**) Transmission electron micrographs showing the dispersion of AgNPs, and (**B**) the chemical characterization of AgNPs analyzed by EDS.

The morphology, size distribution, and particle size of the cardamom–AgNPs could all be determined using TEM. Indeed, the only approach that has been shown to be accurate in measuring nanoparticle size is microscopy. The data describing the dispersion and agglomeration of nanoparticles were collected using TEM imaging. The cardamom–AgNPs produced in this study were found to be monodispersed, mostly spherical, semi-uniformly shaped, and with little aggregation, as shown in the high-resolution TEM micrograph in Figure 4A. Furthermore, this TEM image shows that the cardamom–AgNPs were 10–50 nm in size, with an average particle size of 24.50 nm.

Figure 4B shows the recorded EDS profile used for elemental compositional analysis. The chart showing the percentage weight of each element confirmed the presence of around 66.96% of an AgNP; an optical absorption peak at around 3 keV is often seen in metallic AgNPs due to their SPR [36]. The presence of the platinum (Pt) element in the EDS analysis is attributed to the sample grid used during the analysis. Sodium (Na), magnesium (Mg), silicon (Si), and chlorine (Cl) elements are added to silver metal when conjugated biomolecules are present on the surface of AgNPs [37]. When extracts from plants’ aboveground parts are used in the biosynthesis of cardamom–AgNPs, a multi-dimensional product is produced. The optical and electrical characteristics of the metallic NPs give them a distinct identity, and their fluctuating values make them unique. Previous studies have reported comparable results [38,39,40], describing how the synthesis created spherical nanoparticles devoid of agglomeration and with a single peak at 3 keV, which indicates the presence of silver.

**Figure 5:**
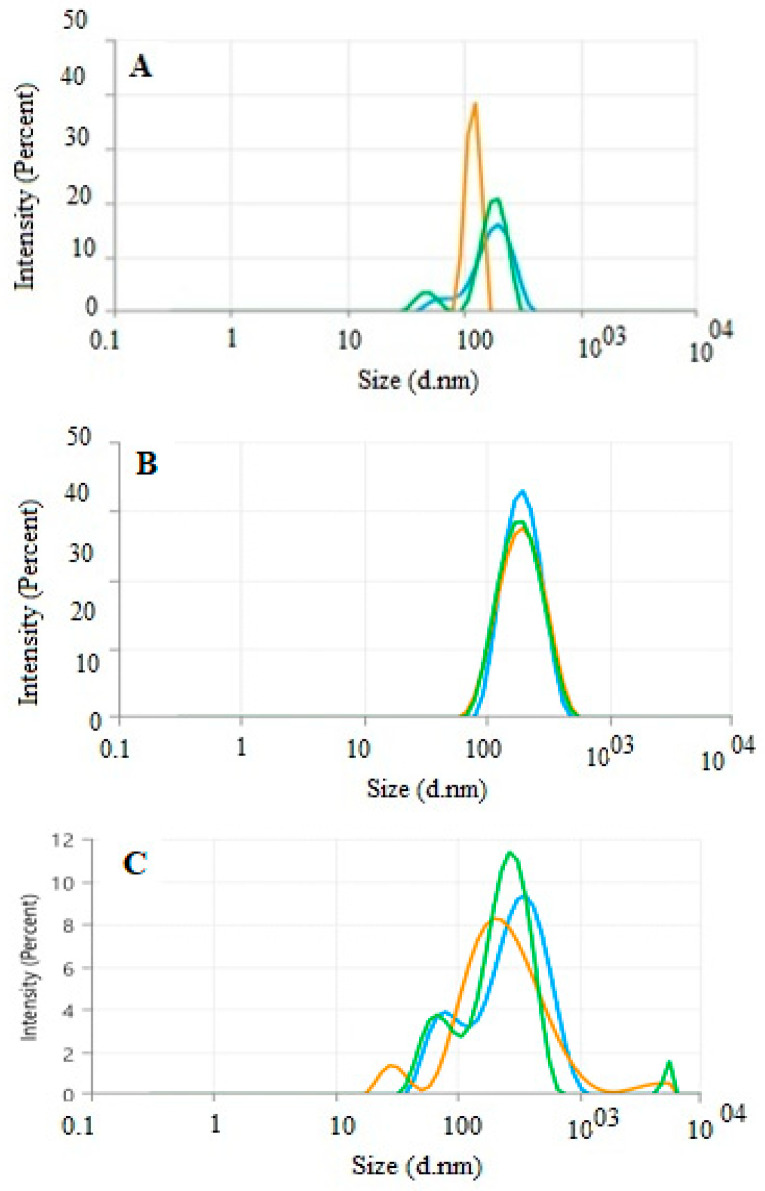
Intensity-based size distribution analysis using DLS technique of *cardamom*–AgNPs with varying concentrations: (**A**) (1.77 mM), (**B**) (3.68 mM), and (**C**) (8.83 mM).

The distribution profiles of AgNP samples synthesized through eco-friendly methods were thoroughly analyzed using a particle size analyzer. Dynamic light scattering (DLS) was utilized to assess the intensity, number, and volume distribution of the nanoparticles. The size distribution by intensity was measured across all *cardamom*–AgNP concentrations. As shown in Figure 5A–C, the average nanoparticle sizes and z-average hydrodynamic diameters were approximately 336.3 nm, 174 nm, and 179.3 nm for concentrations of 1.77, 3.68, and 8.83 mM, respectively. Furthermore, the corresponding polydispersity index (PDI) values were 0.3551, 0.1473, and 0.4421, reflecting the monodispersity, polydispersity, and stability of the synthesized nanoparticles. The size distribution curves A and C show at least two distinct peaks, indicating the presence of multiple particle populations. This confirms that the nanoparticle distribution is not entirely monodisperse at these concentrations. The broader size distributions and multiple peaks observed in these curves may result from particle aggregation, irregular crystallization, or heterogeneous nucleation [41]. In contrast, curve B shows a single sharp peak, indicative of a more uniform and monodisperse nanoparticle population with greater stability [42].

### 3.3. Evaluation of Cytotoxicity

The results in Table 1 demonstrate that the 24 h ATCBRA insecticide and *cardamom*–AgNP treatments had the effect of decreasing the mitotic index compared to the control (MI = 18.7 ± 4.3), unlike *E. cardamomum* aqueous extract treatment. This indicates that the MI was inhibited due to exposure to *cardamom*–AgNPs, with results showing an inverse association between the treatment concentration and MI, as 24 h exposure to 60 mg of *cardamom*–AgNPs lowered the MI to 5 ± 0.8. In contrast, the maximum MI increase relative to the control was observed in the 24 h treatment with a 0.1% concentration of insecticide (24.2 ± 3.5). In other words, while the roots exposed to ATCBRA insecticide achieved the highest mean MI value at a 0.1% concentration, the MI decreased at higher insecticide concentrations; this decrease in MI values is known to be associated with increasing insecticide concentrations. The roots exposed to 4, 5, and 6% concentrations of *E. cardamomum* aqueous extract showed higher MI values than those exposed to 0.3, 0.5, and 0.7% ATCBRA insecticide concentrations, as shown in Table 1. Meanwhile, cardamomum–AgNP treatment showed a significant reduction in MI values in comparison with the cardamom aqueous extract and ATCBRA insecticide treatments, achieving the lowest MI values, while the *E. cardamomum* aqueous extract produced the highest overall MI values in comparison to other treatments.

**Figure 6:**
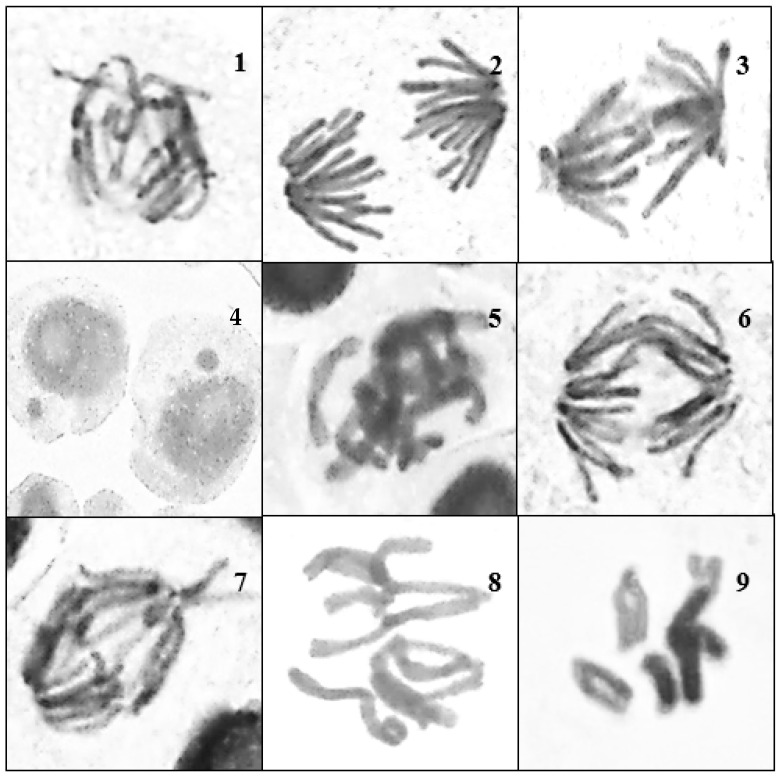
Different types of abnormal mitotic phases seen after treating *Vicia faba* roots with ATCBRA insecticide for 24 and 48 h. 1 = disturbance in the anaphase; 2 = diagonal with fragment anaphase; 3 = diagonal and multi-bridge anaphase; 4 = micronuclei in the telophase; 5 = free chromosome and stickiness in metaphase; 6 = multi-bridges in the anaphase; 7 = multi-bridges and vagrant chromosomes in the anaphase; 8 = disturbance and laggard chromosome in the metaphase; 9 = C-mitosis.

**Figure 7:**
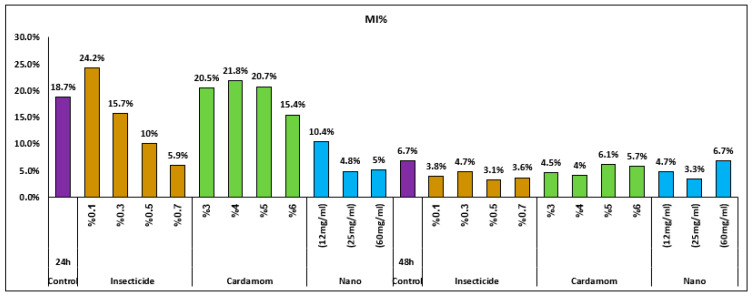
Effect of different treatments TCBRA insecticide, *E. cardamomum* aqueous extract, and *cardamomum*–AgNPs on the mitotic index of *Vicia faba* root tips after 24 and 48 h of exposure.

The MI values obtained for the 48 h treatments were similar, although the 0.5% concentration of insecticide showed the lowest value (3.1 ± 0.4) compared to the control (6.7 ± 1), while the highest MI value among all the other treatments was seen in roots exposed to 60 mg of *cardamom*–AgNPs (6.7 ± 0.9). In comparing the MI values of all treatments, we found that *E. cardamomum* aqueous extract had the highest MI values consecutively and affected *V. faba* roots’ MI least at both 24 h and 48 h exposure times, followed by the cardamom–AgNP treatment, with the lowest MI values observed in roots exposed to ATCBRA insecticide treatment, as shown in Figure 6 and Figure 7.

At 48 h, the mitotic index for cells exposed to the *cardamom*-synthesized AgNPs is not significantly different from that of the control group. This suggests that the AgNPs, under the conditions tested (including the concentration used and an exposure duration of 48 h), do not strongly interfere with cell division processes. In other words, the nanoparticles do not appear to induce cytotoxicity or genotoxicity severe enough to alter the normal frequency of mitosis in root tip cells [43]. The observed differences in the cell division index for *cardamom*-derived silver nanoparticles at 24 and 48 h may be attributed to variations in their chemical properties, bioavailability, and cellular interaction mechanisms. These nanoparticles are synthesized using a plant extract which contains bioactive compounds that can modify their surface charge, hydrophobicity, and oxidative potential. Such modifications may enhance the cytotoxicity and reactivity of the nanoparticles with cellular components. Extract-derived silver nanoparticles may induce oxidative stress, leading to DNA damage, cell cycle arrest, or apoptosis, which can lower the cell division index. Cells exposed to these nanoparticles might recover or adapt over time, resulting in there being no significant difference compared to the control group [44]. Conversely, prolonged exposure to the nanoparticles might cause cumulative damage, though the reduction in the mitotic index may not be proportionate due to the limited viability of the remaining cells. Additionally, the bioactive compounds in cardamom extract may enhance the ability of silver nanoparticles to interfere with cell cycle machinery, such as by disrupting mitotic spindle formation or inhibiting DNA synthesis. Differences in nanoparticle dispersibility, stability in the medium, or cellular uptake efficiency could also contribute to the effects observed at different time points [43,44,45,46].

There are several possible reasons why chromosome aberrations for the insecticide synthetic chemical pesticides show the lowest MI values at a maximum concentration of 0.7%. The insecticide may trigger mechanisms in the cells that stabilize chromosomes or prevent aberrations. There may be reduced bioavailability of the compound at higher concentrations due to aggregation and decreased solubility. Alternatively, impurities or co-factors in the insecticide formulation at this concentration might mitigate its genotoxic effects. Additionally, the 0.7% concentration could represent a threshold where further increases in concentration neither exacerbate genotoxicity nor actively suppress aberrations. This might be due to the saturation of interaction sites or other limiting factors in the system. It is also possible that the active ingredient in the insecticide loses its potency at higher concentrations due to chemical degradation, poor absorption, and other factors that reduce its genotoxic potential [47,48].

### 3.4. Evaluation of Genotoxicity (CA Index)

**Figure 8:**
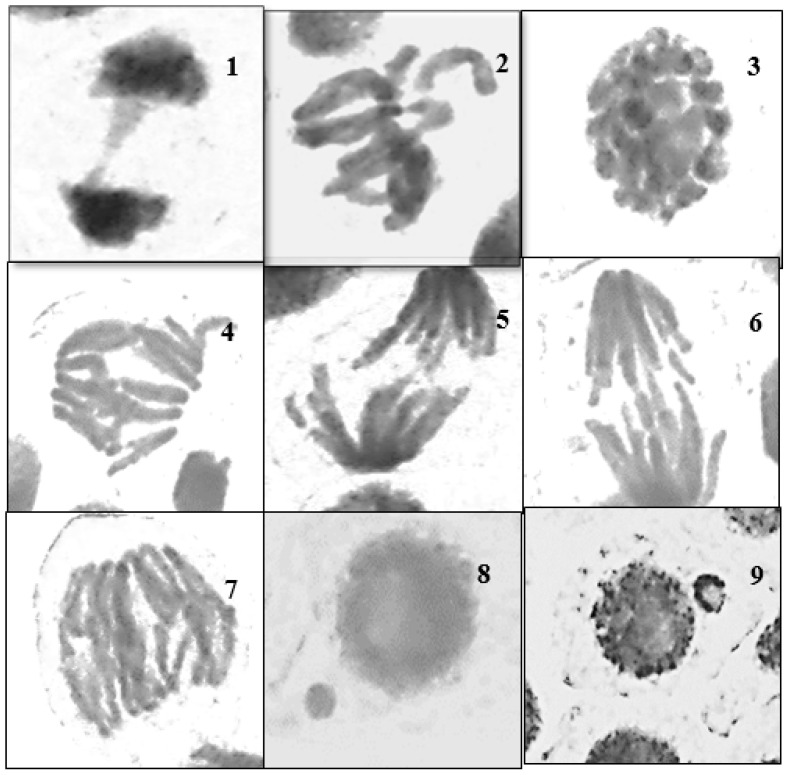
Different types of abnormal mitotic phases after treating *Vicia faba* roots with *Elettaria cardamomum* aqueous extract for 24 and 48 h. 1 = stickiness and bridge in the telophase; 2 = disturbance and laggard chromosome in the metaphase; 3 = sticky prophase; 4 = multi-bridges at anaphase; 5 + 6 = fragment at anaphase; 7 = multi-polar and multi-bridge anaphase; 8 + 9 = micronuclei in the telophase.

**Figure 9:**
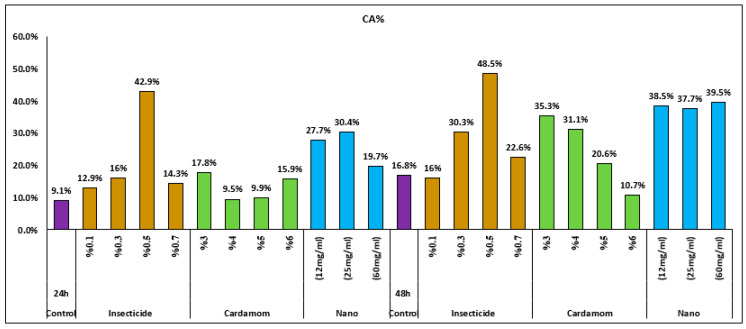
Effect of different treatments (ATCBRA insecticide, *E. cardamomum* aqueous extract, and cardamomum–AgNPs) on chromosomal aberration (CAs) in root tips of *Vicia faba* after 24 and 48 h exposure.

Among all treated *V. faba* roots, those exposed to cardamom–AgNP treatment scored the highest CA values (42.9% at the 0.5% concentration), followed by those exposed to ATCBRA insecticide, while roots exposed to the *E. cardamomum* aqueous extract treatment had the lowest CA rate (Figure 8 and Figure 9 and Table 2).

All dosage levels of the insecticide caused various chromosomal aberrations. Although fragments, multipolarity, C-mitosis, and star metaphase were not detected at all concentrations, the most frequently observed aberrations were disturbance, bridges, stickiness, and laggard/s. There were a number of mitotic defects in cells treated with the cardamom extract, including disruption, bridges, stickiness, and lagging chromosomes, whereas star metaphase and multipolarity were not found at all dosage levels. Finally, root cells that underwent cardamom–AgNP treatment showed various chromosomal aberrations, such as disturbance, bridges, and stickiness, although, as with the previous treatments, fragments and star metaphase were not observed at all concentrations (Figure 6 and Figure 8).

The mitotic index is considered one of the most important cytotoxic parameters; thus, any alteration in the mitotic index is considered a parameter for cytotoxicity. Our study revealed that cardamom aqueous extract treatment was the least effective treatment with regard to the mitotic index. However, at most of the concentrations of this treatment, it scored a higher MI value compared to the control.

Research by Chegini and Abbasipour [49,50] has shown that cardamom essential oil is harmful to the insect *Tuta absoluta*, a major pest targeting the Solanaceae family of plants. There are likely to be significant benefits for farmers, the environment, and human health when cardamom essential oil is used as an all-natural pesticide instead of a synthetic one [51]. However, despite the fact that it is recognized to possess several bioactive qualities, there has been a dearth of research on the topic of cardamom essential oil’s insecticidal action.

Similar to the effect of cardamom, many studies have demonstrated the effectiveness of plant extracts in eliminating insects. For example, volatile oils derived from the Zingiberaceae plant *Etlingera elatior* have a suppressive influence on the mitotic index of *A. cepa* roots [52]. In addition, *Punica granatum* L. peel extract has been suggested as a promising alternative to chemical pesticides and can be used as a biopesticide against larval and adult red palm weevils [53]. Previous results also showed that the leaf extracts of *Juglans regia* have the potential to be used as an efficient pesticide against *Sitophilus oryzae* [54].

On the other hand, ATCBRA insecticide is a commercially formulated mixture of a synthetic pyrethroid insecticide, cypermethrin (3%, *w*/*v*), and an organophosphate insecticide, quinalphos (20%, *w*/*v*), that is commonly used on various agricultural crops [55]. Quinalphos is the most extensively used organophosphate insecticide in agriculture, while cypermethrin is also one of the most widely used insecticides in the world for pest control and crop loss prevention in agriculture. However, as a highly toxic chemical that can be inhaled, ingested, or enter the body dermally, its widespread use can have various toxic effects on non-target organisms [56,57]. In our study, a major reduction in the mitotic index values of root tips exposed to ATCBRA insecticide was noted. Similarly to our results, Karaismailoglu [58] stated that the effect of fipronil insecticide on the MI and the frequency of mitotic phases showed all concentrations of fipronil markedly decreased the MI values of *A. cepa* somatic chromosomes compared to the control. Further, the MI has been shown to decrease with increasing concentrations of the chemical pesticides malathion and carbendazim. ATCBRA insecticide was considered to have a cytotoxic effect on *Vicia faba* root tips [59].

Many of our MI values were lower under insecticide treatment than in the control. The MI-reducing effect of cypermethrin can be explained by different mechanisms, the most important of which is the interruption of the cell cycle as a result of MN and CA formation induced by cypermethrin, which was demonstrated in a previous analysis [60]. Our results confirmed that cypermethrin causes chromosomal aberrations, which is in agreement with Rao et al. [61] while opposing the results of Asita and Makhalemele [62], who reported cypermethrin (Alpha-thrin) was cytotoxic, but not genotoxic, at various concentrations. All concentrations of cypermethrin induced chromosomal abnormalities in the present study, the most common of which were chromosome bridges. Sticky chromosomes are sub-chromatid bridges that attach the two chromatids together [63,64], ensuring the highly toxic effects of insecticides.

Agrochemicals have been shown to impact plant health by inducing genotoxic damage to essential biomolecules, including DNA. This damage is primarily attributed to an increase in the production of reactive oxygen species (ROS), as noted by Sies, 2015 [65]. Reactive oxygen species (ROS), which are formed when agrochemical active ingredients accumulate excessively, cause oxidative and genotoxic damage to fundamental biomolecules in plant cells, which in turn impairs plant health and productivity [66,67,68]. High concentrations of reactive oxygen species (ROS) are highly detrimental to organisms, leading to lipid peroxidation, protein oxidation, and damage to cellular components and nucleic acids. Additionally, ROS can inhibit enzyme activity and disrupt physiological and biochemical processes [69,70].

**Figure 10:**
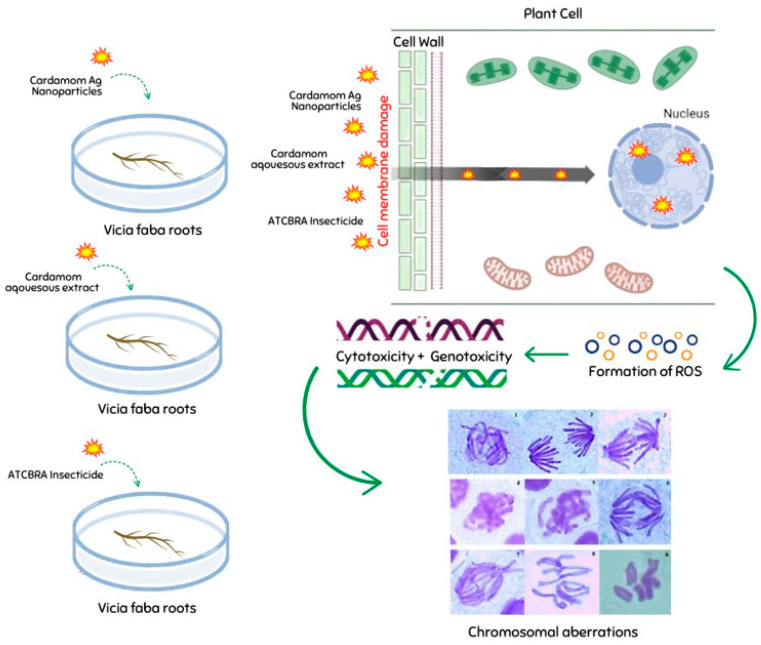
A schematic illustration of the proposed mechanism underlying the cyto-genotoxic effects of biopesticides (including plant extracts), nanoparticles, pesticides in the root model system.

On the other hand, a decrease in mitotic index values was noted in the cardamom–AgNP treatment with increasing exposure time and concentration. Babu et al. [71] found a similar trend in *A. cepa* root tips treated with nano-silver, with a decrease in MI values. Increasing toxicant treatment induced cell death as a consequence of AgNP exposure; it was claimed that the interfering impact of the maximum concentration of cardamom–AgNPs on mitotic activity was due to cells delaying the S phase (DNA synthesis) and stopping the G2 phase. Based on previous studies [72,73], the nano-silver may have reduced the MI values because of its influence on growth frequency via its capacity to inhibit or block the creation of metabolites necessary for a proper mitotic sequence. Figure 10 shows a schematic illustration of the proposed mechanism underlying the cyto-genotoxic effects of the extracts, nanoparticles, or pesticides in our root model system.

## 4. Conclusions

In conclusion, this study successfully synthesized silver nanoparticles (AgNPs) using *Elettaria cardamomum* (cardamom) extracts. GC-MS analysis revealed that the cardamom extract contained bioactive compounds. The green synthesis of AgNPs was confirmed by UV-Vis spectroscopy, which showed a peak between 420 and 430 nm, and by TEM imaging, which demonstrated that the nanoparticles were monodispersed, primarily spherical, and semi-uniform in shape. Energy-dispersive spectroscopy (EDS) further confirmed that cardamom–AgNPs comprised 60.5% of the total weight. Moreover, the z-average hydrodynamic diameters, measured using the DLS technique, were approximately 336.3 nm, 174.0 nm, and 179.3 nm for concentrations of 1.77 mM, 3.68 mM, and 8.83 mM, respectively. Additionally, the corresponding polydispersity index (PDI) values were 0.3551, 0.1473, and 0.4421, reflecting variations in particle uniformity and stability across the different concentrations. The cytotoxic and genotoxic effects of cardamom extract, cardamom–AgNPs, and the insecticide ATCBRA on the root system of *Vicia faba* (broad bean) were assessed using two key indicators: the mitotic index (MI) and chromosomal aberrations (CA). Roots were exposed to varying concentrations of ATCBRA (0.1%, 0.3%, 0.5%, 0.7%), aqueous cardamom extract (3%, 4%, 5%, 6%), and biosynthesized cardamom–AgNPs (12, 25, 60 mg) over 24 h and 48 h periods. Among the treatments, the cardamom aqueous extract demonstrated the least cytotoxicity, as it maintained higher MI values and caused minimal chromosomal aberrations. This suggests there was a mild genotoxic effect with limited interference in mitotic activity. In contrast, *cardamom*-synthesized AgNPs showed a dose-dependent reduction in MI after 24 h, with cytotoxic effects diminishing by 48 h. However, these nanoparticles induced the highest rates of chromosomal aberrations, including dislocations, bridges, and adhesions, which was likely due to oxidative stress and DNA damage caused by the presence of bioactive compounds on their surface. The ATCBRA insecticide exhibited the strongest cytotoxic and genotoxic effects, with MI values significantly decreasing as concentrations increased. It caused extensive chromosomal abnormalities, such as sticky chromosomes and bridges, which we attributed to its chemical composition disrupting cell division. The findings emphasize the need for further research into biosynthesized nanomaterials to better understand their effects, optimize concentrations and treatment durations, and explore their potential applications in promoting plant growth and sustainable pest management. This approach could help to reduce environmental and health risks associated with chemical pesticides, offering a promising direction for more eco-friendly agricultural practices.

## Figures and Tables

**Table 1:** Effect of different treatments (ATCBRA insecticide, *Elettaria cardamomum* aqueous extract, and *cardamomum*–AgNPs) on the mitotic index (MI) of *Vicia faba* root tips after 24 h and 48 h exposure.

Treatments	Exposure Time (h)	Concentration	Mitotic Index MI ± SE
ATCBRA insecticide	24 h	Control	18.7 ± 4.3
		0.1%	24.2 ± 3.5
		0.3%	15.7 ± 2.6
		0.5%	10 ± 0.8
		0.7%	5.9 ± 0.9 *
*Elettaria cardamomum* extract		Control	18.7 ± 4.3
		3%	20.5 ± 2.4
		4%	21.8 ± 1
		5%	20.7 ± 1.3
		6%	15.4 ± 1.3
*Cardamom*–AgNPs		Control	18.7 ± 4.3
		12 mg/mL	10.4 ± 1.5
		25 mg/mL	4.8 ± 0.9 *
		60 mg/mL	5 ± 0.8 *
ATCBRA insecticide	48 h	Control	6.7 ± 1
		0.1%	3.8 ± 1.4
		0.3%	4.7 ± 0.1
		0.5%	3.1 ± 0.4 *
		0.7%	3.6 ± 0.1 *
*Elettaria cardamomum* extract		Control	6.7 ± 1
		3%	4.5 ± 0.7
		4%	4 ± 1.4
		5%	6.1 ± 0.5
		6%	5.7 ± 0.3
*Cardamom*–AgNPs		Control	6.7 ± 1
		12 mg/mL	4.7 ± 1.4
		25 mg/mL	3.3 ± 0.8
		60 mg/mL	6.7 ± 0.8

* = significantly different from the control at the 0.05 significance level (*p* ≤ 0.05).

**Table 2:** Effect of different treatments (ATCBRA insecticide, *Elettaria cardamomum* aqueous extract, and cardamom–AgNPs) on chromosomal aberrations (CAs) in *Vicia faba* root tips after 24 h and 48 h exposure.

Treatments	Exposure Time (h)	Concentration	Chromosomal Aberration % CA ± SE
ATCBRA insecticide	24 h	Control	9.1 ± 2.4
		0.1%	12.9 ± 4.6
		0.3%	16 ± 1.2 *
		0.5%	42.9 ± 2.9 *
		0.7%	14.3 ± 2.3
*Elettaria cardamomum* extract		Control	9.1 ± 2.4
		3%	17.8 ± 4.7
		4%	9.5 ± 1.6
		5%	9.9 ± 2.4
		6%	15.9 ± 4.2
Cardamom–AgNPs		Control	9.1 ± 2.4
		12 mg/mL	27.7 ± 11.4
		25 mg/mL	30.4 ± 3.6 *
		60 mg/mL	19.7 ± 2.5 *
ATCBRA insecticide	48 h	Control	16.8 ± 3.6
		0.1%	16 ± 4.1
		0.3%	30.3 ± 3.1 *
		0.5%	48.5 ± 12.3 *
		0.7%	22.6 ± 9.3
*Elettaria cardamomum* extract		Control	16.8 ± 3.6
		3%	35.5 ± 6 *
		4%	31.1 ± 7.9 *
		5%	20.6 ± 0.6 *
		6%	10.7 ± 1.7
*Cardamom*–AgNPs		Control	16.8 ± 3.6
		12 mg/mL	38.5 ± 7.3
		25 mg/mL	37.7 ± 2.8
		60 mg/mL	39.5 ± 2.1 *

* = significantly different from the control at the 0.05 significance level (*p* ≤ 0.05).

## Data Availability

The data supporting the findings of this study are available.
